# Lightning initiation on the exoplanet K2-18b

**DOI:** 10.1038/s41598-026-57486-2

**Published:** 2026-06-20

**Authors:** Elloïse Fangel-Lloyd, Saša Dujko, Ilija Simonović, Hannah Diamond-Lowe, Sven Karlsson, Christoph Köhn

**Affiliations:** 1 Danish Meteorological Institute, Sankt Kjelds Plads, Copenhagen, Denmark; 2 Department of Space Research and Technology, Technical University of Denmark, Elektrovej, Kgs. Lyngby, Denmark; 3 Institute of Physics, University of Belgrade, Pregrevica, Belgrade, Serbia; 4 Space Telescope Science Institute, 3700 San Martin Drive, Baltimore, MD 21218, USA; 5 Department of Applied Mathematics and Computer Science, Technical University of Denmark, Richard Petersens Plads, Kgs. Lyngby, Denmark; 6 Department of Physics, Technical University of Dortmund, Otto-Hahn-Straße 4, Dortmund, Germany

**Keywords:** Lightning, Exoplanet, K2-18b, Streamer, Astronomy and planetary science, Climate sciences, Planetary science

## Abstract

Recent JWST data provide a basis for investigations into exoplanetary atmospheres at novel high precision. Weather and climate on these planets, along with the implications of the observed atmospheric composition for extraterrestrial life, remain hotly debated. Using photochemical modelling data based on JWST observations as inputs for a thoroughly validated plasma simulation code, we present the first simulations of streamers, precursors of lightning, in an exoplanetary atmosphere. We find the electrical breakdown field magnitude in this atmosphere to be approximately half that of Earth, indicative of an atmosphere more susceptible to lightning than our own. We show that lightning can incept on K2-18b in all three tested atmospheric configurations, and that variations in atmospheric composition, in particular in the presence of water vapour, affect the rate of streamer inception.

**Supplementary Information:**

The online version contains supplementary material available at https://doi.org/10.1038/s41598-026-57486-2.

## Introduction

Thunderclouds tend toward a particular structure: updrafts carry ice crystals upwards, where they collide with graupel and transfer charge, such that the ice crystals gain a net positive charge and the graupel a net negative charge^1–3^. Their relative difference in weight, along with the tendency to accumulate positive charge on the lower rim of the cloud due to higher temperatures and precipitation, create an electrical tripole in sufficiently tall thunderclouds: typically positive at the top and bottom, and negative in the middle^4,5^. (Stolzenburg et al.^6^ provided a rigorous overview of prevailing theories and relevant measurements on thunderstorm electric field structure.) Balloon experiments have determined this thunderstorm electric field to have magnitudes significantly below the breakdown electric field $E_k$^7–9^, which specifies the field at which a medium is transformed from a dielectric into a conducting plasma^2^.

The *cumulonimbus* thundercloud forms when a *cumulus* cloud transitions into *cumulus congestus*, which may itself then grow into a mature thundercloud^2,10^. These clouds are powerful convection cells, transporting heat and moisture over large distances. Cooray^2^ described the presence of sufficient water vapour for the growth of thunderclouds on Earth, but lightning is also observed in other atmospheres. Lightning has been detected on Jupiter^11,12^, whose atmosphere is hydrogen-dominated^13^; similarly, Uranus^11^ and Saturn^14^ have also been observed to host lightning. While previous work has discussed the presence of water clouds in other atmospheres (e.g. on Jupiter^15–17^), a rigorous study should consider the possibility of lightning initiation mechanisms that are independent of the presence of water clouds. Hodosán et al.^18^ and Hodosán et al.^19^ discussed lightning as a potential source of radio emissions observed from the exoplanet HAT-P-11b, and Helling et al.^20^ discussed similar emissions observed in various extrasolar brown dwarfs. While atmosphere and cloud composition play a major role in thundercloud formation, there remain gaps in our understanding of lightning propagation in other atmospheres.

Previous work has demonstrated that lightning may have formed the basis for life on Earth. The Miller-Urey experiment^21–23^ found that laboratory spark discharges could produce amino and carbon acids important for biological processes in the atmosphere of Primordial Earth. Köhn et al.^24^ simulated discharges in the Miller-Urey mixtures, finding that lightning may have been easier to incept than on today’s Earth. In situations where observation is impractical, such simulations can provide insight into thunderstorm dynamics, as well as the effect of lightning on atmospheric chemistry and composition.

### Lightning inception

Lightning initiation on Earth is widely understood to begin with streamer inception, when an avalanche of rapid electron ionisation transitions into a plasma channel^2,25^, the so-called streamer. At later stages, streamers heat up, forming the hot and long leaders^26^ which are observed as the bright lightning discharge. During the initial electron avalanche, sufficient spatial separation between the fast electrons and the heavy ions results in the additional space charge electric field, giving rise to further ionisation. The avalanche and subsequent streamer development requires the ambient electric field to surpass the breakdown field $E_k$, defined as the field strength at which electrons experience impact ionisation and attachment in equal measure. Laboratory experiments find $E_k = 3.2$ MV m$^{-1}$ for atmospheric air at standard temperature and pressure (*p=1* bar, *T=300* K)^27^. The scalar $E_k$ is heavily dependent on dynamics at the molecular level, and thus on the ambient gas density $n_{amb}$; decreasing $n_{amb}$ results in decreased $E_k$^28–30^.

The breakdown field $E_k = 3.2$ MV m$^{-1}$ is significantly larger than observed ambient thunderstorm fields $E_{amb}$, which have been measured to assume values on the order of $E_{amb} = 0.3$ MV m$^{-1}$ at ground pressure^7,25^. In isolation, this finding would suggest that the thunderstorm field is too weak to cause electrical breakdown, and thus that lightning inception via the suggested mechanism is entirely impossible. This conflict is resolved by examining the local electric field, rather than the ambient thunderstorm electric field, which may span multiple kilometres. Local perturbations to the electric field, such as raindrops^25^ and ice crystals^9,31^ suspended in clouds can enhance the magnitude of the local electric field to values exceeding $E_k$^32–34^. The accumulation of dense charge regions arising during rapid ionisation^2^distorts the field further; electrons are fast-moving relative to the positive ions created through impact ionisation^2,35^, and the spatial separation between pockets of positive and negative charge result in a space charge electric field **E**$_{space}$. At all times, the total electric field **E**$_{total}$ is given by

1 $$\begin{aligned} \mathbf{E}_{total} = \mathbf{E}_{amb} + \mathbf{E}_{space} \end{aligned}$$

For the avalanche-to-streamer transition, the space charge field must take on values $|\textbf{E}_{space}| \geqslant |\textbf{E}_{amb}|$^36,37^, increasing the rate of ionisation further and leading to the formation of a self-sustaining plasma channel. As such, this transition is highly dependent on the resulting superposition of local perturbations onto the ambient thunderstorm field.

The avalanche-to-streamer transition represents a fundamental step, as lightning inception and propagation hinges on the accumulation of dense charge regions and subsequent amplification of the local electric field^2,24^. In the context of modelling lightning inception, we study streamer inception, the initiation point.

Whilst negative streamers grow through the diffusion of electrons out of the streamer head against the direction of the electric field and electron impact ionisation, the development of positive streamers (propagating against the direction of electron drift^38^) requires a secondary ionisation mechanism^2,38^. In Earth’s atmosphere this role is filled by photoionisation^2,24,39,40^, where electrons excite molecular nitrogen, emitting an ultraviolet photon which then ionises molecular oxygen. The propagation of positive streamers in anoxic laboratory environments is observed^41^, but the source of free electrons may be unclear in less-usual gas mixtures. Even if the source is known, developing a parameterisation and implementing this into a simulation model may not be trivial.

The avalanche-streamer mechanism for lightning inception is widely accepted for Earth’s atmosphere, and is also believed to be the cause of lightning on other bodies. Gibbard et al.^42^ modelled charge separation on Jupiter, and a more recent model presented by Janalizadeh and Pasko^13^simulated streamer inception on Jupiter by including the effects of an external magnetic field into a streamer model. Köhn et al.^43^ simulated streamer inception on Titan, again using the electron avalanche-streamer transition as a basis. Dubrovin et al.^41^ performed laboratory experiments in gas mixtures approximating those of Venus and Jupiter, observing streamer discharges to occur. While imaging techniques can observe lightning strikes on planets within the solar system (e.g Magalhães and Borucki and Kolmašová et al.^44,45^ describe observations of lightning on Jupiter), exoplanets such as K2-18b are too remote for these methods^18^.

### Meteorological conditions on extrasolar bodies

The sub-Neptune planetary class consists of bodies whose mass lies between $M_{\oplus }$ and $M_\psi$; their atmospheres, if present, are hydrogen-dominated.^46^ This term refers to exoplanets, as there do not exist sub-Neptunes in our solar system.^46^ More recently, the concept of a “Hycean” world, consisting of an ocean surface and a hydrogen-rich atmosphere, was introduced^47^ and discussed^48–50^ in connection with the James Webb Space Telescope (JWST)^51^ providing new data with higher precision. Central to these investigations was the question of habitability, and the search for extraterrestrial life.^52^

Hubble, Spitzer and Kepler telescope data presented opportunities to understand the atmosphere of the exoplanet K2-18b^53–55^ prior to the availability of JWST data. In examining the observed Hubble spectra, Benneke et al.^56^ and Tsiaras et al.^57^ discussed water clouds as a potential explanation for detections of water vapour on K2-18b. Methane is also discussed as a likely source of JWST signals previously attributed to water vapour.^49,53^ More recent models with JWST data applied climate models to K2-18b; Barrier and Madhusudhan^58^ found deep convection to occur exclusively in the lower atmospheric levels, and noted that convection is required for the development of *cumulonimbus* thunderclouds.

Charge separation within the thundercloud system arises in the presence of strong updrafts, which transport light particles upwards, and gravity, which pulls heavier particles downwards.^2,3,59,60^ K2-18b has mass *M = 8.63* $M_{\oplus }$^61^, with Benneke et al.^56^ finding *g = 12.4* m s$^{-2} = 1.26$ $g_{\oplus }$; stronger gravitational acceleration may be presumed to result in increased downward movement from heavier graupel. We may carefully speculate that this increased gravitational pull could give rise to an increased charge separation and potentially increased charge density in the generated dipole, which would presumably lead to greater atmospheric electrification. While this hypothetical is interesting, we do not test it in the current work.

This work anticipates the presence of Earth-like thunderstorms, where thunderstorms arise from electric fields occurring in sufficiently tall clouds and lightning incepts via the streamer-leader mechanism. Storm system modelling and forecasting on Earth often considers the Convective Available Potential Energy (CAPE) (e.g Dowdy and Catto^62^.), a measure of the potential for upward motion;^2,10,63^ high CAPE values are linked with severe weather^10,63,64^ and increased lightning flash rates.^65,66^ Barrier and Madhusudhan^58^ report modelled CAPE values on K2-18b in excess of 3 kJ kg$^{-1}$, which is more than sufficient for the development of large thunderstorms on Earth.^2,67,68^ For comparison, atmospheric sounding data analysed by Tuluri et al.^69^ found that the highest observed CAPE value during the 2005 Category 5 Hurricane Katrina was 2.81 kJ kg$^{-1}$.

### Lightning outside of the solar system

Previous work in the exoplanetary lightning literature spans various approaches. Sergeev et al.^70^ used a storm-resolving climate model with a lightning parameterisation on the tidally locked terrestrial exoplanet TRAPPIST-1e, discussing the effects of surface pressure (among others) on lightning flash rate. Braam et al.^71^ simulated lightning-induced chemistry on Proxima Centauri b, again a tidally locked terrestrial exoplanet and via Earth-like thunderstorm mechanisms; Ardaseva et al.^72^ modelled such chemistry for more general atmospheric compositions. Laboratory experiments with spark discharges, such as those described by Barth et al.^73^, may also be used to describe extrasolar environments.

Whether there is sufficient atmospheric electrification to generate lightning on K2-18b is unknown at present (and, given practical considerations of in-situ experiments, likely to remain unknown for some time); this necessitates the assumption that thunderstorm mechanisms operate in the same manner as on Earth and that thunderstorm electric fields develop similarly. Yair^59^ discussed both a lack of available data and gaps in our understanding of charge separation processes in ice as barriers to accurate theories of atmospheric electricity on other planets; it is concluded that applying models of electrification of Earth-like clouds to other planets would require many free parameters to be assumed.^59^ Here, we use models based on the recent JWST data to provide an atmospheric composition and assume, from arguments discussed above, that background electrification can occur.

Wogan et al.^74^ presented modelling results for three possible scenarios of K2-18b: an input atmosphere with trace CH$_4$, corresponding to an uninhabited Hycean world (hereafter scenario A), an atmosphere corresponding to an inhabited Hycean world (scenario B), and a regime assuming chemical equilibrium and no habitable surface, corresponding to a mini-Neptune (scenario C). These compositions are shown in Tables 1, 2. Photochemical modelling was carried out using these scenarios as a basis by Cooke and Madhusudhan^75^.

**Table 1:** Breakdown fields $E_k$ for various gas mixtures: The first row shows the reduced field $E_k/n_{amb}$ in units of Td. The second row shows $E_k$ for an ambient gas number density $n_{amb} = 2.5047 \times 10^{25}$ m$^{-3}$. $E_k$ values for Earth are taken from^24^.

	Pure H$_2$	Scenario A	Scenario B	Scenario C	Earth
$E_k/n_{amb}$ [Td]	35.1	65.3	68.6	65.9	126
$E_k$ [MV m$^{-1}$]	0.879	1.64	1.72	1.65	3.20

**Table 2:** Mixing ratio of relevant gas mixtures. All values are given in %.

	Scenario A	Scenario B	Scenario C
H$_2$	90.65	87.4	86.992
H$_2$O	8.5	10	8.1
CH$_4$	2.0 $\times 10^{-8}$	1.8	4.32
CO$_2$	0.8	0.8	0.088
CO	0.05	2.65 $\times 10^{-3}$	0.44
NH$_3$	-	-	0.06

Given these three potential atmospheric compositions, we aim to study lightning in these atmospheres. We proceed by first calculating the breakdown field $E_k$. Determining $E_k$is not in itself a novel approach; for example, Helling et al.^12^ determined breakdown quantities in extrasolar brown dwarfs. We here follow the procedure described by Köhn et al.^24^, where $E_k$ is calculated from simulated electron attachment and ionisation rates, and subsequently used to inform the ambient electric field strength imposed in streamer simulations.

With these streamer simulations we aim to understand whether streamers can incept via an Earth-like mechanism, where ambient electrification combined with local perturbations to the electric field give rise to streamer propagation. By choosing three potential atmospheres for study, we also aim to discuss the sensitivity of lightning initiation to atmospheric composition. While the precision of observational data has greatly improved following JWST results, there remain significant uncertainties in the exact composition of these faraway atmospheres. Understanding the sensitivity of the system may also provide an interesting physical context to our obtained results.

## Results

### The breakdown field

**Fig. 1:**
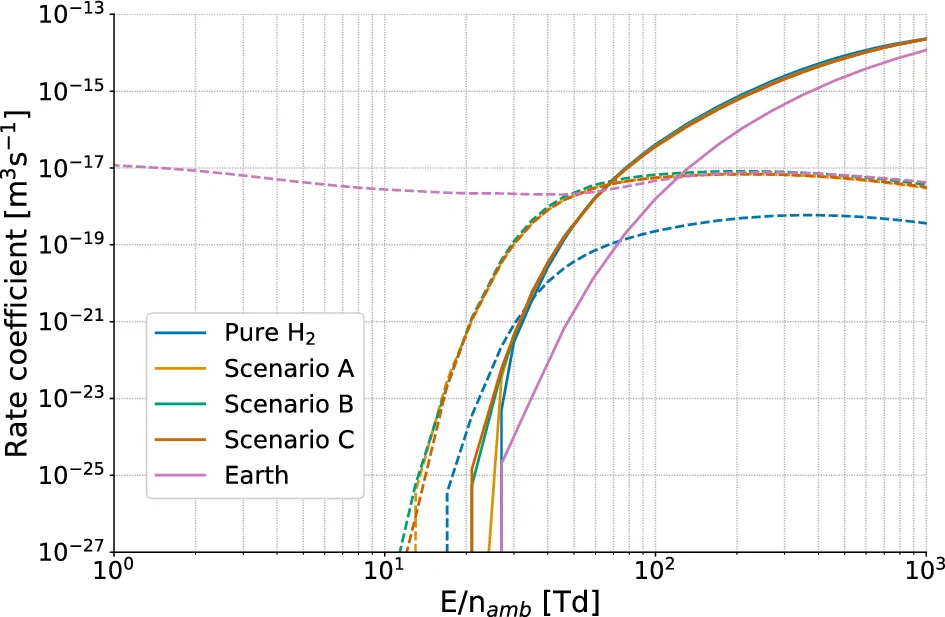
Attachment and ionisation rates as a function of reduced electric field $E/n_{amb}$ for the three K2-18b scenarios, along with pure H$_2$ and Earth (with the dry-air assumption common to streamer contexts, 80% N$_2$ and 20% O$_2$). $n_{amb}$ is the ambient gas density. Dashed lines show attachment rates and solid lines ionisation rates. Other calculated transport data are made available, please see the declarations.

In order to calculate $E_k$, we first determine ionisation and attachment rates; for detail, please see Methods. Figure 1 shows the rates of ionisation and attachment (*α* and *η*, respectively, see Eq. (4)) in each of the scenarios, along with pure molecular hydrogen and a modern Earth environment. For fields *⩾ 100* Td, the ionisation rates are very similar for all considered scenarios, except for Earth. The attachment rate of H$_2$ differs consistently from the rates of the other mixtures, as all gases in the simulated K2-18b atmosphere are attaching. The modern Earth scenario has non-zero attachment for all energies 1 eV $\leqslant \epsilon \leqslant 10^3$ eV; this is due to the occurrence of three-body attachment to molecular oxygen, which is not present in the exoplanet mixtures.

The breakdown field $E_k$ is defined as the field at which ionisation and attachment occur in equal measure, and is here determined for the three scenarios, along with that of pure hydrogen (for comparison purposes).

Table 1 shows breakdown field values calculated from simulation data (procedure as described by Li et al.^76^). The pure H$_2$ case has a very low breakdown field due to its very few excitational modes. The three tested K2-18b scenarios are similar in magnitude, with Scenario B being the highest, implying that the electron avalanche and subsequent streamer transition is slightly harder to achieve in these conditions. All three K2-18b scenarios find breakdown fields significantly lower than that of Earth, due to their high proportion of H$_2$, indicating atmospheres which may be more receptive to lightning development than our own.

### The evolution of streamer discharges

**Fig. 2:**
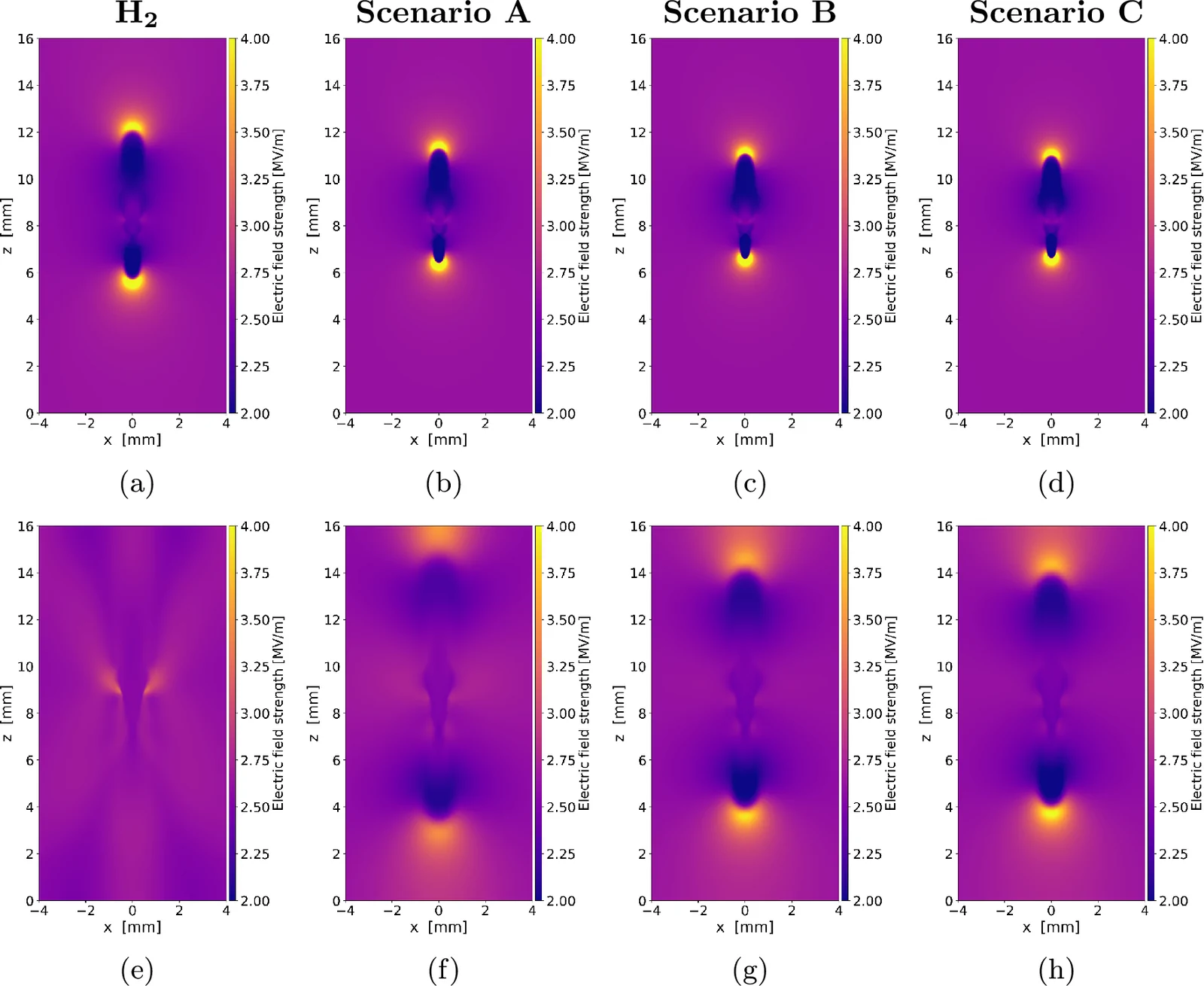
Electric field strength for the three test cases (Tab. 2), along with a pure H_2_ atmosphere. The top row shows data at *t = 6.5* ns, and the bottom row at *t = 9.5* ns. From left to right, the columns show: pure H_2_, Scenario A, Scenario B, Scenario C. Electron density plots are provided in Supplement 1. The ambient field $E_{\text {amb}}=2.625$ MV m$^{-1}$ is oriented in negative *z*-direction. The domain has been clipped in the *x*-dimension for viewing clarity.

Figure 2 shows streamer development in the three gas mixtures summarised in Table 2, along with a molecular hydrogen test case. Positive streamers are propagating downwards and negative streamers upwards, all in a reduced electric field of 105 Td. As this field strength is larger than the breakdown fields (Table 1), streamers incept in all considered cases. At *t = 6.5* ns, the electric field profiles of the streamers appear very similar in the three K2-18b scenarios, but the H$_2$ scenario is markedly more advanced, with positive and negative tips further extended from the origin. At *t = 9.5* ns, more distinct differences arise: the streamer in atmosphere (A) is longer and more diffuse than in (B) and (C). The H$_2$ test case is significantly further advanced, and is approaching the *x*-boundaries; this can also be observed in the electron density plots presented in Supplement 1.

Streamers, as modelled here, are found to be possible in the environment of K2-18b. The rate at which they incept differs, however, with streamers in Scenario A growing faster than the other tested K2-18b atmospheres, and pure hydrogen incepting rapidly. This is consistent with the finding that of the possible K2-18b atmospheres, the breakdown field of Scenario A is lowest (see Tab. 1). The calculated friction force, a measure of deceleration experienced as a result of collisions, is provided in Supplement 2.

## Discussions

We present calculated breakdown electric fields for three potential K2-18b atmospheres, and observe that $E_k$ is approximately half that of Earth. This suggests that a significantly lower level of sustained thunderstorm electrification is necessary for lightning inception than on Earth.

In addition, this work presents the first simulations of streamer inception as a precursor of lightning on an extrasolar body, and a material step towards a thorough understanding of atmospheric electricity in sub-Neptunes, using K2-18b as a test case. Streamer simulations performed in three configurations of atmospheric composition for the exoplanet K2-18b have found that streamers can incept in all scenarios; as such, lightning is possible, and potentially highly probable, in this environment. This, along with the low breakdown fields observed, motivate the conclusion that K2-18b is likely to experience increased atmospheric electrification and higher lightning flash rates than we do on Earth (if lightning occurs). In their applications of lightning parameterisation models to Proxima Centauri b and TRAPPIST-1e, respectively, Braam et al.^71^ and Sergeev et al.^70^ found lower flash rates than observed on Earth. However, our findings are consistent with those of Barrier and Madhusudhan^58^, who have found that the meteorological conditions conducive to thunderstorms on K2-18b do arise. We note that Proxima Centauri b and TRAPPIST-1e are considered terrestrial exoplanets,^70,71^ and inhabit a different atmospheric parameter space than the sub-Neptune K2-18b, whose atmosphere is H$_2$-dominated. We conclude that lightning storms are highly likely on this exoplanet, and may occur far more frequently than on Earth. Other sub-Neptunes with H$_2$-rich atmospheres and the potential for convection may also experience lightning; future studies in this arena should consider the possibility of lightning on these planets.

Streamer inception occurs more quickly in the Scenario A atmosphere, presumably due to its lower breakdown field. It is observed that the presence of other gases in a H$_2$ mixture can affect the rate of streamer inception, even when H$_2$ accounts for $\geqslant 90\%$ of the mixing ratio. We posit that the Scenario B atmosphere experiences a higher breakdown field because of its high water vapour content; water vapour is noted to have very large collision probabilities, and so causes much energy loss in colliding electrons. Despite the Scenarios B and C having very similar H$_2$ concentrations, their $E_k$ differ significantly; thus, we conclude that the described techniques are more sensitive to water vapour content than to concentrations of other gases. We do not observe particular sensitivity to methane content. The breakdown fields of the three K2-18b scenarios differ by no more than 5%, a similar magnitude to variations in gas composition. Streamers incept rapidly in the pure H$_2$ atmosphere, and the breakdown field for H$_2$ is less than one-third of Earth’s; this is consistent with the findings of Dubrovin et al.^41^, who noted that streamer propagation occurred more easily in the hydrogen-rich Jovian atmosphere than in Earth-like mixtures because hydrogen has very few excitational modes. Hydrogen-rich atmospheres may be likely candidates for lightning detection in future.

Our code does not include thermal effects and so cannot simulate the streamer-leader transition and thus the full lightning strike;^2^ codes capable of self-consistently modelling these physics are not found in the literature, as this is an extremely computationally demanding task. However, we have studied streamer inception as the initiation point for lightning, as is common throughout the atmospheric electricity community, and find that lightning can incept.

Previous work has linked lightning to the generation of molecules necessary for life^21–23^ and discussed the effect of lightning on atmospheric composition.^73,77^ The question of K2-18b’s habitability is beyond the scope of this work; however, studies on this subject may benefit from studying the role played by electrical discharges in this planet’s atmosphere. In the future, we aim to analyse the chemical changes caused by electricity in such atmospheres, and study whether lightning can play a major role in the habitability of K2-18b.

## Methods

### A fluid code for streamer discharges

A fluid-based streamer code, which assumes continuous electron and ion densities across the computational domain,^38,78,79^ is applied. The time evolution of the system is modelled by tracing the electron ($n_e$), positive ion ($n_p$) and negative ion ($n_n$) particle number densities [m$^{-3}]$.

An ambient electric field $\textbf{E}_{amb}$ is imposed across the domain, representing the electric field generated by the thunderstorm system; this field is assumed spatially uniform and constant in time. In addition, local perturbations to the ambient field arise when dense charge regions experience a spatial separation (due to higher mobilities in electrons than ions^2^); the total electric field is determined by solving Poisson’s equation for the electric potential

2 $$\begin{aligned} \nabla ^2 \phi = -\frac{q_e(n_p-n_e-n_n)}{\epsilon _0} \end{aligned}$$

given the elementary charge $q_e$ and the permittivity of vacuum $\epsilon _0$. The total electric field $\textbf{E}$ is then given by $\textbf{E}=-\mathbf {\nabla } \phi$. Perturbations to this field affect the electron drift velocity $\textbf{W}(\textbf{r}, t)$

3 $$\begin{aligned} \textbf{W}(\textbf{r}, t) = \mu \ \textbf{E}(\textbf{r}, t) \end{aligned}$$

with the electron mobility *μ* and diffusion tensor D($\textbf{r}, t)$.

This numerical scheme for simulating both positive and negative streamers advances in time by solving the drift-diffusion-reaction equations (4) using the above terms. If photoionisation is present in a given atmosphere, a source term $S_{ph}$ is included;^39,40,76^ we do not have sufficient data to model photoionisation in K2-18b’s atmosphere self-consistently, and so it is neglected here. As a secondary source of ionisation is required for positive streamer propagation,^2,40^ we follow the technique described by Köhn et al.^24^ and use an initial level of background ionisation. In Earth’s atmosphere, there exists a level of near-constant ionising radiation caused by radioactive decay processes and cosmic rays.^24,80^ In-situ measurements of background ionisation levels on extrasolar bodies are likely to remain impossible for some time. The background ionisation values in Earth’s atmosphere $n_B$ are found to be $\approx 10^9 - 10^{10}$ charged particles per cubic metre.^80,81^ Wormeester et al.^81^ find that increasing $n_B$ to larger values, such as $n_B = 10^{13}$ m$^{-3}$, causes more rapid streamer growth, and note that background ionisation levels in Earth’s atmosphere increase to this level in the event of pulsed repetitive discharges, i.e. repeated lightning strikes. To facilitate rapid inception of the positive streamer, and thus avoid long simulation runtimes, we use $n_B = 1 \times 10^{13}$ m$^{-3}$. Advances in computational science may in the future allow for methods where these assumptions are not necessary; we use them here to permit the simulation of positive streamers.

4 $$\begin{aligned} \begin{aligned} \frac{\partial n_e}{\partial t} + \nabla \cdot (n_e \textbf{W} - \text {D} \nabla n_e)&= n_e (\alpha - \eta ) \ |\textbf{W}|,\\ \frac{\partial n_p}{\partial t}&= n_e \alpha \ |\textbf{W}|,\\ \frac{\partial n_n}{\partial t}&= n_e \eta \ |\textbf{W}|. \end{aligned} \end{aligned}$$

With $\alpha (\textbf{r}, t)$ and $\eta (\textbf{r}, t)$, the ionisation and attachment rate coefficients, respectively.

To represent continuous quantities on a discrete numerical grid, a finite volume method is applied. Density and potential variables are defined at cell centres and electric field variables at cell faces. To avoid numerical diffusion or oscillations, the Koren flux limiter^82^ is applied to the advective term of the electron flux equation. A thorough description is provided by Simonović et al.^83^

Time-stepping takes place via a second-order Runge-Kutta method. Three time step restriction criteria are applied; the most significant of these is the Courant-Friedrichs-Lewy (CFL) condition for the advection-diffusion equation

5 $$\begin{aligned} \Delta t \left( \sum _{i=1}^{2} \frac{|v_i|}{\Delta x_i} + 2 \sum _{i=1}^{2} \frac{D_e}{\Delta x_i^2} \right) < \text {CFL} \end{aligned}$$

with velocity components $v_i$, grid spacing $\Delta x_i$ along dimension *i*, the diffusion coefficient $D_e$ and CFL are chosen values ensuring stability (for further detail, see Simonović et al.^83^). Other imposed limitations on the time step arise from the dielectric relaxation time and the rates of non-conservative processes; they are not presented here, but are discussed by Simonović et al.^83^.

In the interest of computational efficiency, the code employs adaptive mesh refinement provided by the AMReX^84^ library. Refinement criteria and implementation details are provided by Simonović et al.^83^. A geometric multigrid solver^84^ is applied to solve Poisson’s equation (2).

A full overview of code validation and benchmarks against the Afivo program^85^ is presented by Simonović et al.^83^ Initial conditions corresponding to positive streamers in air are tested,^83,86^ as well as negative streamers in strongly attaching mixtures of CO$_2$ and C$_4$F$_7$N. Very good agreement is found for all test cases; relative differences to the Afivo code do not exceed 5%.

### Determining the breakdown field

Fluid codes such as those described above do not accept cross-section data as direct model inputs, but rather, electron transport data. These transport data are often obtained by first performing swarm simulations, where electrons are permitted to fly and collide with an ambient medium using Monte Carlo techniques.^87^ Köhn et al.^24^ and Li et al.^76^ also analyse transport data calculated via this technique.

By tracking the impact ionisation and attachment events of individual electrons, ionisation and attachment rates as those shown in Figure 1 can be calculated. The breakdown field $E_k$ is defined as the field at which ionisation and attachment occur in precisely equal measure,^2,27^ which can then be obtained from these rate coefficients straightforwardly.

### Model inputs and initial conditions

Table 2 shows an overview of the possible atmospheric compositions of K2-18b used as model inputs. These data are taken from figures presented by Wogan et al.^74^, assuming an atmospheric pressure of 1 bar, which Wogan et al.^74^ denoted the model surface. We note that JWST observations of K2-18b are generally constrained to the 0.1 - 10 mbar range;^49,88^ surface pressure in modelling approaches vary, see e.g Madhusudhan et al.^48^., where a surface pressure of 100 bar is used. While the atmospheric compositions described by Wogan et al.^74^ are naturally pressure-dependent, streamer physics do not depend on pressure. Instead, the physical quantities governing streamer growth scale with $E/n_{amb}$, such that a higher electric field is required to incept streamers in higher-density mixtures.^9^ Simulating streamer inception at higher altitude results in lower $n_{amb}$, and thus in lower $E_k$; we simulate at the ground pressure suggested by Wogan et al.^74^, and we anticipate that this is where streamers are hardest to incept.

The initial conditions are identical in each tested scenario; the physical domain is represented in 2D axi-symmetric space by axial (*z*) and radial (*r*) components, with domain size *z = 16* mm and *r = 16* mm. Simonović et al.^83^ discussed chosen cross-section data for electron scattering. The initial electron and positive ion densities are described by a Gaussian patch

6 $$\begin{aligned} n_{e,p} = n_B + n_0 \exp \left( -\frac{(z - z_0)^2 + r^2}{\sigma ^2}\right) \end{aligned}$$

with width *σ = 0.2* mm, maximum density $$n_0 = 5 \times 10^{18}$$ m$$^{-3}$$, origin $$r_0, z_0 = (0,8.0)$$ mm and $$n_B = 1 \times 10^{13}$$ m$$^{-3}$$ as discussed above. The ambient electric field imposed across the domain $$\textbf{E}_{amb} = 2.625$$ MV m$$^{-1}$$ (*∼ 105* Td for $$n_{amb} = 2.5047 \times 10^{25}$$m$$^{-3})$$ is oriented in the negative *z* direction. This field corresponds to $$\approx 1.5 E_k$$ for the Scenario B gas composition mixture. The ambient field $$E_{amb} = 1.5 E_k$$is ubiquitous throughout streamer literature (see e.g^24,35,43^.); this choice facilitates shorter runtimes and more rapid streamer inception. While positive streamers may incept at for $$|\textbf{E}_{amb}| < E_k$$^24,25^, negative streamers require $$|\textbf{E}_{amb}| > E_k$$ in order to incept and propagate.^2,38^

## Supplementary Information

Supplementary Information 1.

Supplementary Information 2.

## Data Availability

Calculated transport data are made available via the following DOI https://doi.org/10.5281/zenodo.17395338.
