# Supplementary material for: Lightning initiation on the exoplanet K2-18b
Source: Sci Rep. 2026 Jun 20;16:28287. doi: 10.1038/s41598-026-57486-2 (PMC13558627; doi:10.1038/s41598-026-57486-2)
Supplement: Supplementary file 1 [file 41598_2026_57486_MOESM1_ESM.pdf]

Supplement 1

Supplementary Figure 1 - Electron density data

Figure 1 shows electron density plots for the same times and scenarios as Fig. 2. As above, the streamer in H_2 develops most quickly, followed by the Scenario A streamer.

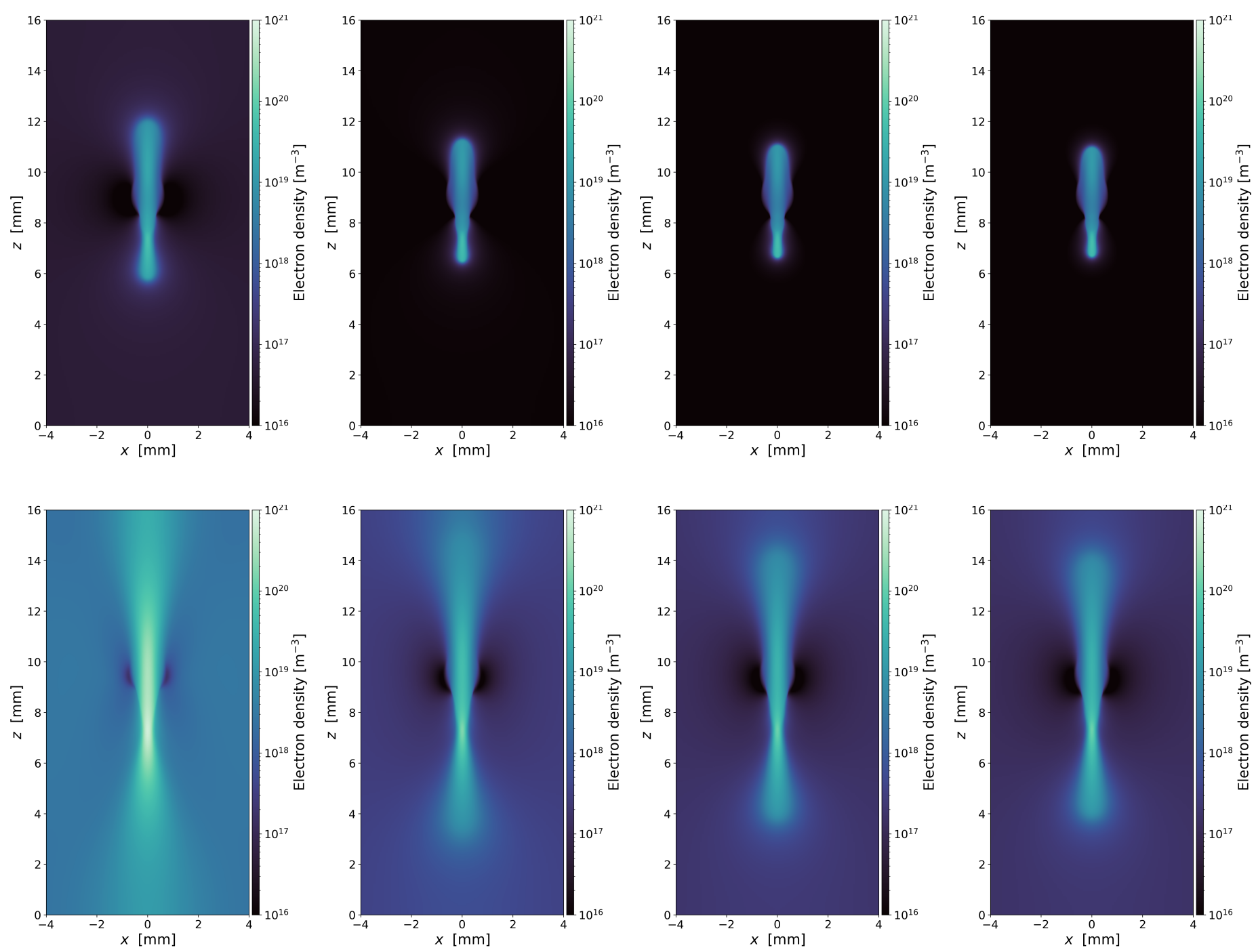

Figure 1: Electron number density for the three test cases, along with a pure H_2 atmosphere. The top row shows data at $t = 6.5$ ns, and the bottom row at $t = 9.5$ ns. From left to right, the columns show: pure H2, Scenario A, Scenario B, Scenario C. The ambient field $E_{amb} = 2.625$ MV m^{-1} is oriented in negative z-direction.
