# Supplementary material for: Lightning initiation on the exoplanet K2-18b
Source: Sci Rep. 2026 Jun 20;16:28287. doi: 10.1038/s41598-026-57486-2 (PMC13558627; doi:10.1038/s41598-026-57486-2)
Supplement: Supplementary file 2 [file 41598_2026_57486_MOESM2_ESM.pdf]

Supplement 2

Supplementary Figure 2 - The friction force

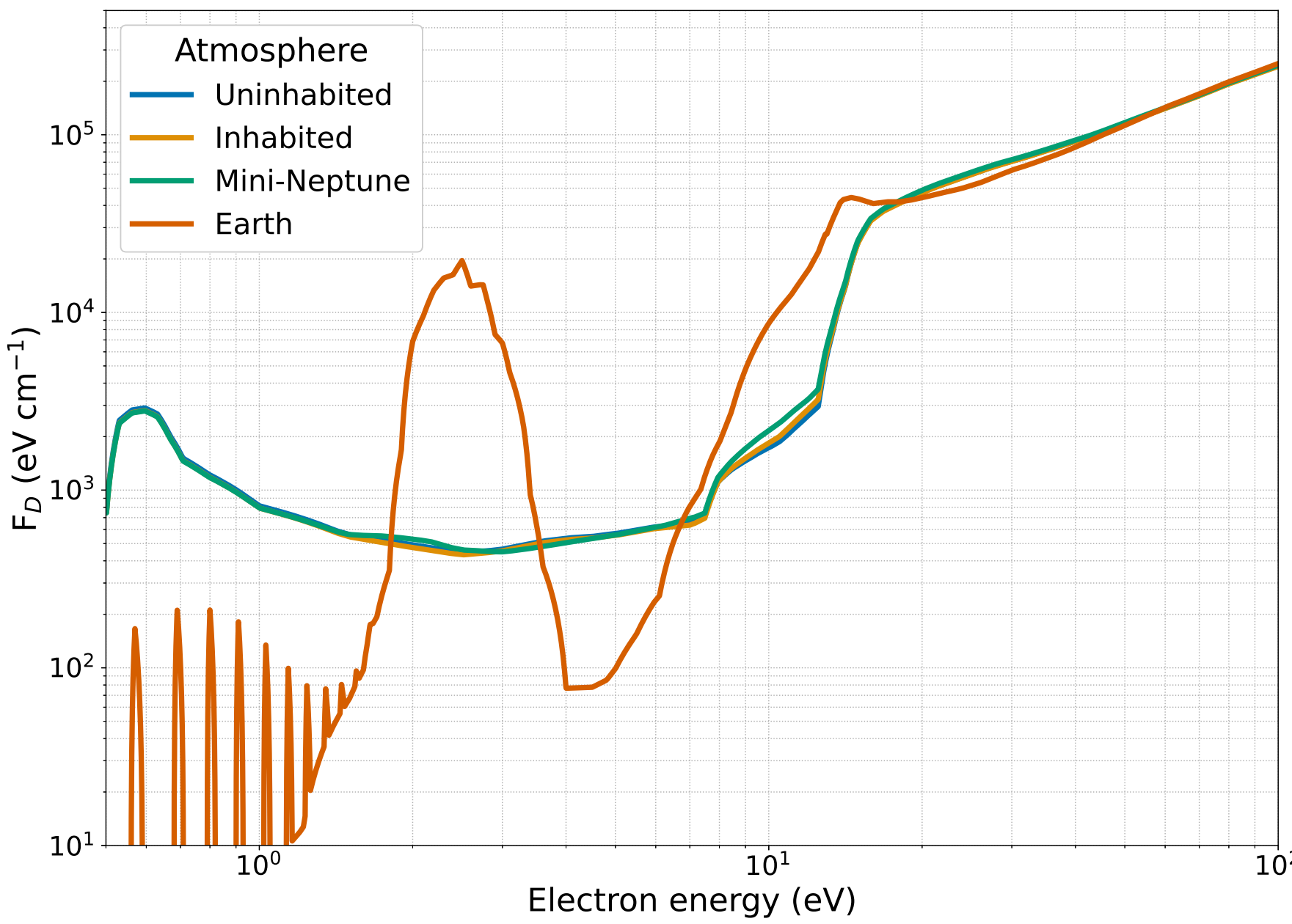

Figure 1: Friction force $\mathbf{F}_D$ calculated for three possible K2-18b atmospheres, alongside a dry-air Earth atmosphere.

Supplementary Note

Figure 1 shows the friction force for an electron in the three tested gas mixtures simulating the atmosphere of K2-18b. The friction force is calculated as in [2], by summing over the jth collision cross-section

$$\mathbf{F}_D(\epsilon) = \sum_j n_j \ \sigma_j(\epsilon) \ \Delta\epsilon_j \tag{1}$$

with n_j the partial number density of the cross-section, $\sigma_j(\epsilon)$ the cross-section value for an energy ϵ, and $\Delta\epsilon_j$ the energy loss occurring in collision j.

Only inelastic processes are included. As in [1], uniform energy distribution following an ionisation collision is assumed, so the energy loss for ionisation collisions is

$$\Delta\epsilon_j = \frac{1}{2}\left(\epsilon - \epsilon_b\right)$$

with ϵ_b the binding energy of the gas.

A thorough discussion of the dry-air Earth case is given in [2]; it is included in Fig. 1 only for comparison purposes. The friction force experienced by an electron in the atmosphere of K2-18b is considerably higher than in air for $\epsilon <\sim 2$ and $4 < \epsilon < 7$ eV; the sharp local maximum observed in air around 2 eV is not present in K2-18b, due to the absence of these vibrational modes in N_2 and O_2. For $\epsilon > 11$ eV, the friction is similar to that of Earth.

Only in the energy range $7 < \epsilon < 11$ can there be observed a difference between the three tested K2-18b cases, with Scenario C having a slightly higher $\mathbf{F}_D$, followed by Scenario B. This may be due to the relatively large cross-sections for electronic excitation in CH_4 in this energy range, which is most abundant in the Scenario C test case and least abundant in Scenario A.

References

[1] Christoph Köhn et al. "Streamer propagation in the atmosphere of Titan and other N2:CH4 mixtures compared to N2:O2 mixtures". In: *Icarus* 333 (2019), pp. 294–305. ISSN: 0019-1035. DOI: https://doi.org/10.1016/j.icarus.2019.05.036. URL: https://www.sciencedirect.com/science/article/pii/S0019103518307863.

[2] Gregory D. Moss et al. "Monte Carlo model for analysis of thermal runaway electrons in streamer tips in transient luminous events and streamer zones of lightning leaders". In: *Journal of Geophysical Research: Space Physics* 111.A2 (2006). DOI: https://doi.org/10.1029/2005JA011350. eprint: https://agupubs.onlinelibrary.wiley.com/doi/pdf/10.1029/2005JA011350. URL: https://agupubs.onlinelibrary.wiley.com/doi/abs/10.1029/2005JA011350.
